# Dr. John Byrne

**Published:** 1902-11

**Authors:** 


Dr. John Byrne, of Brooklyn, died at Montreaux, Switzerland, October 1, 1902, aged 77 years. He was one of the founders of Long Island College Hospital and organised St. Mary's Female Hospital. He also was one of the founders of St. Mary's General Hospital and served as president of the staff as well as chief surgeon of the institution from its foundation. Dr.

Byrne was a gynecologist of wide reputation, and was a former president of the American Gynecological Society. He was identified with the treatment of uterine cancer by electro-cautery, in the use of which he was expert and wrote many monographs on the subject. He is survived by a widow and two daughters.

